# 

**Published:** 1849

**Authors:** 


### CONTENTS.

### PART I.—ORIGINAL COMMUNICATIONS.

ART. 1.—Cases of Malignant Pneumonia, By Edward R. Parks, M.D. 461
ART. 2.—Remedy for Deafness, by C. Brackett, M.D. 467
ART. 3.—Malformation of the Heart, by John Jackson, M.D. 469
ART. 4.—Remarks on Belladonna, by E. S. Cooper, M. D. 471
ART. 5.—Influence of disordered functons of the Skin, by J. Jackson, M.D. 475
ART. 6.—History of a case of Spinal Disease, by Dr. Irwin, 477
ART. 7.—Valedictory Address, by D. Brainard, M. D. 480
ART. 8.—Treatment of Spina Bifidia, by D. Brainard, M.D. 495

### PART II.—REVIEWS AND NOTICES OF NEW WORKS.

ART. 1.—Essays on Infant Therapeutics, by John B. Beck, M.D. 498
ART. 2.—Report on Cholera, by the Sanitory Committee of Board of Health of New York, 498
ART. 3.—Report of Superintendent of Indiana Hospital for Insane, 501
ART. 4.—Report of Trustees of Illinois Hospital for the Insane, 503
ART. 5.—Introductory Lectures, 504
ART. 6.—Text Book of Practical Anatomy, by R. Harrison, M.D. 509
ART. 8.—Medical Lexicon, by D. Meredith Reese, M.D. 510
ART. 8.—Annual Circular of Geneva College, 511
ART. 9.—Lectures on Theory and Practice of Physic, by Bell and Stokes, 511
ART. 10.—A system of Clinical Medicine, by R. J. Graves, M.D. 512

### PART III.—SELECTIONS.

ART. 1.—Passage of an Iron Rod through the head, 523
ART. 2.—Cholera in New Orleans, 519
ART. 3.—Poisoning with Chloride of Zinc, by T. Stratton, M.D., 521
ART. 4. Preparation of Collodion, 525
ART. 5.—Vapor baths in Cholera, 527
ART. 6.—Chloroform and its dangers, 527
ART. 7.—Hood's Illustration of Hydropathy. 530

### PART IV.—EDITORIALS.

ART 1.—Letter from Dr. Hall, 531
ART 2.—Convention of Western Medical Schools, 532
ART. 3.—Department of Medicine in the University of Michigan, 533
ART. 4.—Imposition in the sale of Medicine, 533
ART. 5.—To our Patrons, 534
ART. 6.—Ottawa Medico-Chirurgical Society, 535
ART. 7.—Trial for Mal-practice, 536
ART. 8.—Philadelphia Association for Medical Instruction, 545
ART. 9.—Annual Commencement, 547
ART. 10.—Rush Medical College, 548
ART. 11:—Editorial Changes, 549
ART: 12:—Medical Intelligence, 549

